# Outcome of inguinal hernia repair after previous radical prostatectomy: a registry-based analysis with 12,465 patients

**DOI:** 10.1007/s10029-022-02635-5

**Published:** 2022-06-22

**Authors:** M. Trawa, H. C. Albrecht, F. Köckerling, H. Riediger, D. Adolf, S. Gretschel

**Affiliations:** 1 Department of General, Visceral, Thoracic and Vascular Surgery, Brandenburg Medical School, University Hospital Ruppin-Brandenburg, Fehrbelliner Str. 38, 16816 Neuruppin, Germany; 2 Faculty of Health Brandenburg, Brandenburg Medical School, Neuruppin, Germany; 3 Department of Surgery, Hernia Center, Academic Teaching Hospital of Charité Medical School, Vivantes Humboldt-Hospital Berlin, Berlin, Germany; 4 StatConsult GmbH, Magdeburg, Germany

**Keywords:** Inguinal hernia, Lichtenstein, Laparo-endoscopic, Prostatectomy, Herniamed register

## Abstract

**Introduction:**

Following radical prostatectomy, the rate of inguinal hernias is fourfold higher compared to controls. Laparo-endoscopic repair after previous radical prostatectomy is considered complex. Therefore, the guidelines recommend open Lichtenstein repair. To date, there are limited data on inguinal hernia repair after prior prostatectomy.

**Methods:**

In a retrospective analysis from the Herniamed Registry, the outcomes of 255,182 primary elective unilateral inguinal hernia repairs were compared with those of 12,465 patients with previous radical prostatectomy in relation to the surgical technique. Furthermore, the outcomes of laparo-endoscopic versus open Lichtenstein repair techniques in the 12,465 patients after previous radical prostatectomy were directly compared.

**Results:**

Comparison of the perioperative complication rates for primary elective unilateral inguinal hernia repair with and without previous radical prostatectomy demonstrated for the laparo-endoscopic techniques significantly higher intraoperative complications (2.1% vs 0.9%; *p* < 0.001), postoperative complications (3.2% vs 1.9%; *p* < 0.001) and complication-related reoperations (1.1% vs 0.7%; *p* = 0.0442) to the disadvantage of previous prostatectomy. No significant differences were identified for Lichtenstein repair. Direct comparison of the laparo-endoscopic with the open Lichtenstein technique for inguinal hernia repair after previous radical prostatectomy revealed significantly more intraoperative complications for TEP and TAPP (2.1% vs 0.6%; *p* < 0.001), but more postoperative complications (4.8% vs 3.2%; *p* < 0.001) and complication-related reoperations (1.8% vs 1.1%; *p* = 0.003) for open Lichtenstein repair.

**Conclusion:**

Since there are no clear advantages for the laparo-endoscopic vs the open Lichtenstein technique in inguinal hernia repair after previous radical prostatectomy, the surgeon can opt for one or the other technique in accordance with their experience.

## Introduction

In a population-based nationwide study an almost fourfold increase in groin hernia repair was observed after radical prostatectomy compared with controls [1]. The incidence of inguinal hernia after open radical prostatectomy was 13.7%, after laparoscopic radical prostatectomy 7.5% and after robotic radical prostatectomy 7.9% [2].

Laparo-endoscopic repair of inguinal hernia following radical prostatectomy is considered in the guidelines to be a complex surgical procedure [3–10]. Therefore, the guidelines recommend the open Lichtenstein mesh technique for repair of inguinal hernia following radical prostatectomy [6–10]. Following radical prostatectomy, only highly experienced surgeons should perform a laparo-endoscopic procedure for treatment of inguinal hernia [3–5, 9, 10]. A systematic review [11] with inclusion of 5 feasibility studies with 277 inguinal hernia repairs after previous radical prostatectomy [12–16] demonstrated that experienced minimally invasive surgeons were able to repair such complex inguinal hernias, too, in laparo-endoscopic technique. No statistically significant difference was found in the postoperative complication rate or the recurrence rate between the post-radical prostatectomy group and the control group. Only a significantly higher rate of intraoperative complications was identified due to bleeding from the inferior epigastric vessels [11]. The authors criticize the fact that the sample size reported on in the systematic review was too small to permit proper assessment, stating that further studies on this topic were needed. Since frequently performed previous operations, such as open or minimally invasive radical prostatectomy, are recorded in the Herniamed Registry, it was possible to analyze the patients as a subgroup [17, 18].

## Methods

Herniamed is an internet-based hernia registry in which hospitals and independent surgeons in Germany, Austria and Switzerland can voluntarily enter data on their routine hernia operations [17, 18]. A contract is made with every participating hospital and every participating surgeon where the latter two parties commit to ensuring complete and correct entry into the Herniamed Registry of all data on hernia repairs. However, in order for a patient to be included in the Herniamed Registry, the patient must sign a special consent form agreeing to data documentation and follow-up by the treating hospital or surgeon. If this special consent form is not available the patient must not be documented in the Herniamed Registry. As part of the information provided to patients regarding participation in the Herniamed Registry, they are also told to inform the treating hospital or surgeon about any problems or complications occurring after hernia repair. If problems or complications occur after the operation, the patient can at any time contact the treating hospital or surgeon to request clinical examination [17, 18].

All intraoperative and postoperative complications as well as the complication-related reoperations are recorded for up to 30 days after the operation.

After 1, 5 and 10 years, all patients and their general practitioner are sent a questionnaire by the treating hospital or treating surgeon asking them about any pain at rest, pain on exertion, chronic pain requiring treatment or any protrusion in the groin area or recurrence. Patients are also asked again whether they have experienced any postoperative complications. If the patient or general practitioner reports a relevant finding, the patient may be requested to attend for further diagnostic examination [17, 18]. Haapaniemi et al. [19] could show that participation in the registry and follow-up by a questionnaire and selective physical examination provides a solid basis for quality control.

To demonstrate the influence of previous radical prostatectomy on the outcome of inguinal hernia surgery, data prospectively collected data in the Herniamed Registry between 2010 and 2019 were retrospectively analyzed under different aspects.

1. First, the outcomes of primary elective unilateral inguinal hernia repair in men with and without previous radical prostatectomy were compared for the years 2010 to 2019. Here, only the laparo-endoscopic totally extraperitoneal patch plasty (TEP) technique and the transabdominal preperitoneal patch plasty (TAPP) technique were compared with the open Lichtenstein technique. The aim was to identify the influence exerted by previous radical prostatectomy on the outcome of primary elective unilateral inguinal hernia repair for the laparo-endoscopic and open Lichtenstein techniques.
2. In another analysis, the outcomes of primary elective unilateral inguinal hernia repair following previous radical prostatectomy of the years 2010 to 2019 were directly compared in relation to the laparo-endoscopic surgical techniques versus the open Lichtenstein techniques.
3. To identify differences in time of treatment and perioperative outcome the registry data were also analyzed separately for the years 2010 to 2019. Since the number of repairs recorded in the Herniamed Registry for the years 2010 to 2012 was still relatively low, and the differences relatively large, statistical analyses for treatment and perioperative outcome were carried out for the years 2013 to 2019.

All statistical analyses were performed using the software SAS 9.4 (SAS Institute Inc., Cary, NC). Fisher’s exact test was used for statistical calculations. All single test results for homogeneity between methods are Bonferroni adjusted (factor 2) for multiple testing, thus each *p* ≤ 0.05 represents a significant result in an explorative sense.

## Results

**Fig. 1:**
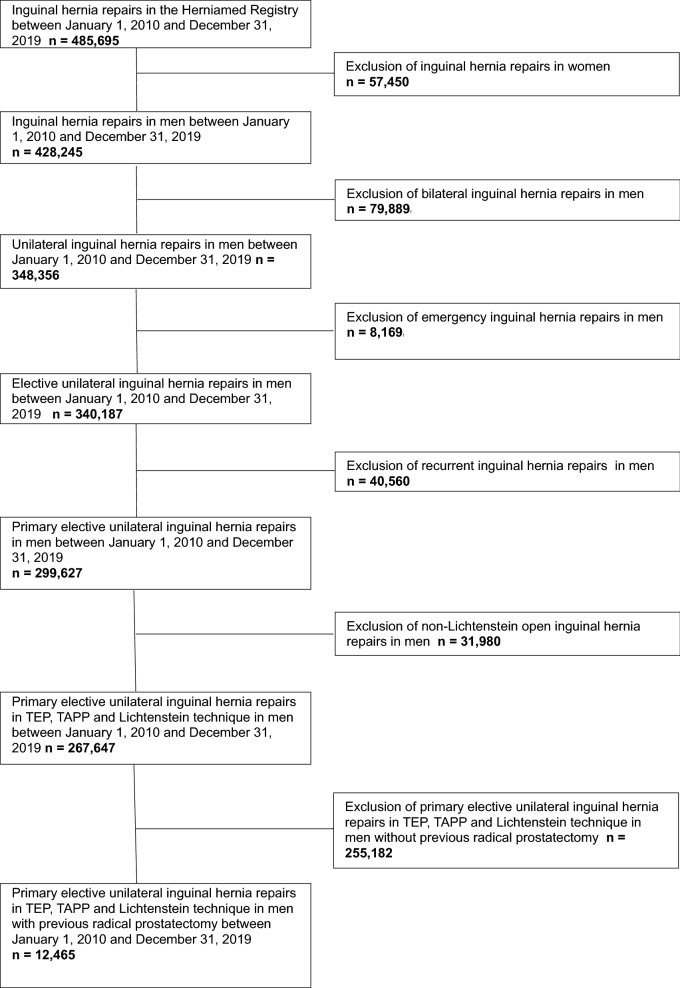
Flowchart of patient inclusion

Between 1 January 2010 and 31 December 2019, 485,695 routine inguinal hernia repairs were documented in the Herniamed Registry (Fig. 1). After subtraction of the female patients, emergency cases, bilateral hernias, recurrences and the open non-Lichtenstein repairs, there remained 267,647 primary elective unilateral inguinal hernia repairs in TEP, TAPP and Lichtenstein technique for subsequent analysis (Fig. 1). These related to 12,465 patients with previous radical prostatectomy and 255,182 patients without previous radical prostatectomy (Fig. 1), corresponding to a proportion of 4.7%.

Inguinal hernia was identified more commonly after open (*n* = 7650/12,465; 61.4%) than after minimally invasive (*n* = 4.819/12.465; 38.6%) previous radical prostatectomy.

**Fig. 2:**
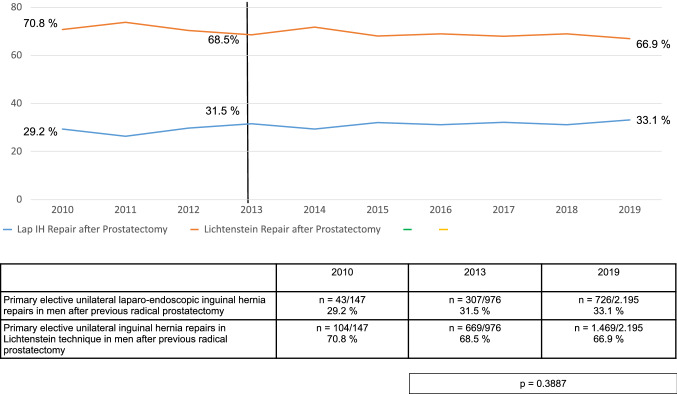
Primary elective unilateral inguinal hernia repairs in men in laparo-endoscopic or Lichtenstein technique following radical prostatectomy (2010–2019) (*n* = 12,465)

Throughout the entire study period two-thirds of all primary elective unilateral inguinal hernia repairs in men after previous radical prostatectomy were carried out in open Lichtenstein technique and one-third in laparo-endoscopic technique (Fig. 2).

Comparison of the outcomes of primary elective unilateral inguinal hernia repair in men after previous radical prostatectomy with those without previous operation revealed significantly more perioperative complications after radical prostatectomy for the laparo-endoscopic techniques (Table 1).

**Table 1:** Perioperative outcome of primary elective unilateral inguinal hernia repair in men in TEP, TAPP and Lichtenstein technique with and without previous radical prostatectomy (2010–2019) (*n* = 267,647)

	Intraoperative complication rates	Postoperative complication rates	Complication related reoperation rates
Primary elective unilateral laparo-endoscopic inguinal hernia repairs in men without previous radical prostatectomy	*n* = 1.554/170.817 0.9%	*n* = 3.203/170.817 1.9%	*n* = 1.221/170.817 0.7%
Primary elective unilateral laparo-endoscopic inguinal hernia repairs in men after previous radical prostatectomy	*n* = 83/3.902 2.1%	*n* = 123/3.902 3.2%	*n* = 41/3.902 1.1%
	*p* < 0.001	*p* < 0.001	*p* = 0.0442
Primary elective unilateral inguinal hernia repairs in Lichtenstein technique in men without previous radical prostatectomy	*n* = 648/84.365 0.8%	*n* = 3.637/84.365 4.3%	*n* = 1.383/84.365 1.6%
Primary elective unilateral inguinal hernia repairs in Lichtenstein technique in men after previous radical prostatectomy	*n* = 53/8.563 0.6%	*n* = 408/8.563 4.8%	*n* = 153/8.563 1.8%
	*p* = 0.8628	*p* = 0.2964	*p* = 1.0000

For example, the intraoperative complications were 2.1% vs 0.9% (*p* < 0.001), postoperative complications 3.2% vs 1.9% (*p* < 0.001) and the complication-related reoperations 1.1% vs 0.7% (*p* = 0.044).

By contrast, no significantly higher perioperative complication rates were identified for open Lichtenstein repair after previous radical prostatectomy compared to those without previous operation.

Further analysis to identify the reason for the higher intraoperative complication rates revealed for the laparo-endoscopic technique after previous radical prostatectomy significantly more bladder and vascular injuries (Table 2). The postoperative complications included more cases of secondary bleeding and seroma (Table 3).

**Table 2:** Details of intraoperative complications of laparo-endoscopic or open Lichtenstein inguinal hernia repairs in men with and without previous radical prostatectomy (2010–2019) (*n* = 267,647)

	Primary elective unilateral laparo-endoscopic inguinal hernia repairs in men without previous radical prostatectomy	Primary elective unilateral laparo-endoscopic inguinal hernia repairs in men after previous radical prostatectomy	*p*	Primary elective unilateral inguinal hernia repairs in Lichtenstein technique in men without previous radical prostatectomy	Primary elective unilateral inguinal hernia repairs in Lichtenstein technique in men after previous radical prostatectomy	*p*
Vessel injury	*n* = 397/170.817 0.2%	*n* = 19/3.902 0.5%	0.0078	*n* = 58/84.365 0.07%	*n* = 12/8.563 0.1%	0.0696
Bowel injury	*n* = 132/170.817 0.08%	*n* = 3/3.902 0.08%	1.0000	*n* = 49/84.365 0.06%	*n* = 5/8.563 0.06%	1.0000
Bladder injury	*n* = 116/170.817 0.07%	*n* = 26/3.902 0.7%	< 0.001	*n* = 32/84.365 0.04%	*n* = 4/8.563 0.05%	1.0000
Nerve injury	*n* = 9/170.817 0.005%	*n* = 1/3.902 0.03%	0.4044	*n* = 245/84.365 0.3%	*n* = 15/8.563 0.2%	0.1064
Others	*n* = 331/170.817 0.2%	*n* = 12/3.902 0.3%	0.2752	*n* = 159/84.365 0.2%	*n* = 16/8.563 0.2%	1.0000

**Table 3:** Details of postoperative complications of laparo-endoscopic or open Lichtenstein inguinal hernia repairs in men with and without previous radical prostatectomy (2010–2019) (*n* = 267,647)

	Primary elective unilateral laparo-endoscopic inguinal hernia repairs in men without previous radical prostatectomy	Primary elective unilateral laparo-endoscopic inguinal hernia repairs in men after previous radical prostatectomy	*p*	Primary elective unilateral inguinal hernia repairs in Lichtenstein technique in men without previous radical prostatectomy	Primary elective unilateral inguinal hernia repairs in Lichtenstein technique in men after previous radical prostatectomy	*p*
Bleeding	*n* = 1.419/170.817 0.8%	*n* = 62/3.902 1.6%	< 0.001	*n* = 2.220/84.365 2.6%	*n* = 263/8.563 3.1%	0.0368
Bowel injury	*n* = 80/170.817 0.05%	*n* = 4/3.902 0.1%	0.2380	*n* = 23/84.365 0.03%	*n* = 3/8.563 0.04%	1.0000
Wound healing disorder	*n* = 180/170.817 0.1%	*n* = 5/3.902 0.1%	1.0000	*n* = 249/84.365 0.3%	*n* = 23/8.563 0.3%	1.0000
Seroma	*n* = 1.497/170.817 0.9%	*n* = 53/3.902 1.4%	0.0062	*n* = 1.221/84.365 1.4%	*n* = 120/8.563 1.4%	1.0000
Infection	*n* = 133/170.817 0.08	*n* = 3/3.902 0.08%	1.0000	*n* = 241/84.365 0.3%	*n* = 29/8.563 0.3%	0.7962
Ileus	*n* = 62/170.817 0.04%	*n* = 4/3.902 0.1%	0.1206	*n* = 19/84.365 0.02%	*n* = 1/8.563 0.01%	1.0000
Others	*n* = 0/170.817 0%	*n* = 0/3.902 0%	1.0000	*n* = 0/84.365 0%	*n* = 0/8.545 0%	1.0000

Direct comparison of the laparo-endoscopic with the open Lichtenstein technique for primary elective unilateral inguinal hernia repair in men after previous radical prostatectomy showed a significantly more unfavorable intraoperative complication rate for TEP and TAPP (2.1% vs 0.6%; *p* < 0.001), but a significantly better postoperative complication rate (3.2% vs 4.8%; *p* < 0.001) and rate of complication-related reoperations (1.1% vs 1.8%; *p* = 0.003) (Table 4).

**Table 4:** Perioperative outcome of primary elective unilateral inguinal hernia repair in men in TEP, TAPP and Lichtenstein technique after previous radical prostatectomy (2010–2019) (*n* = 12,465)

	Intraoperative complication rates	Postoperative complication rates	Complication-related reoperation rates
Primary elective unilateral laparo-endoscopic inguinal hernia repairs in men after previous radical prostatectomy	*n* = 83/3.902 2.1%	*n* = 123/3.902 3.2%	*n* = 41/3.902 1.1%
Primary elective unilateral inguinal hernia repairs in Lichtenstein technique in men after previous radical prostatectomy	*n* = 53/8.563 0.6%	*n* = 408/8.563 4.8%	*n* = 153/8.563 1.8%
	*p* < 0.001	*p* < 0.001	*p* = 0.0034

**Fig. 3:**
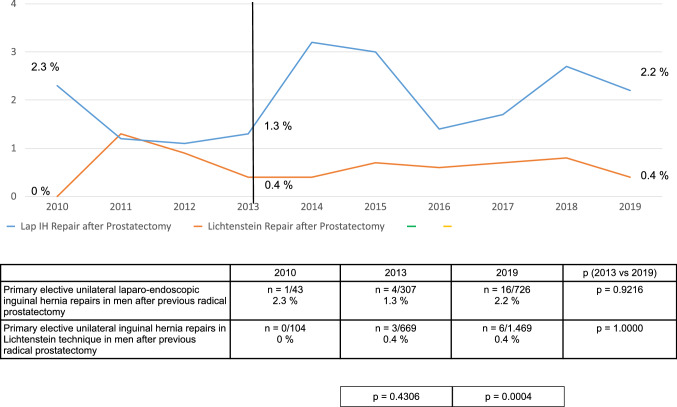
Intraoperative complication rates of primary elective unilateral inguinal hernia repairs in men in laparo-endoscopic or Lichtenstein technique following radical prostatectomy (2010–2019) (*n* = 12,465)

**Fig. 4:**
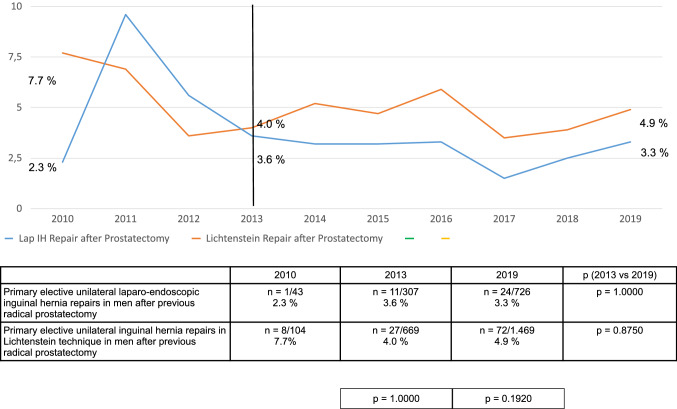
Postoperative complication rates of primary elective unilateral inguinal hernia repairs in men in laparo-endoscopic or Lichtenstein technique following radical prostatectomy (2010–2019) (*n* = 12,465)

**Fig. 5:**
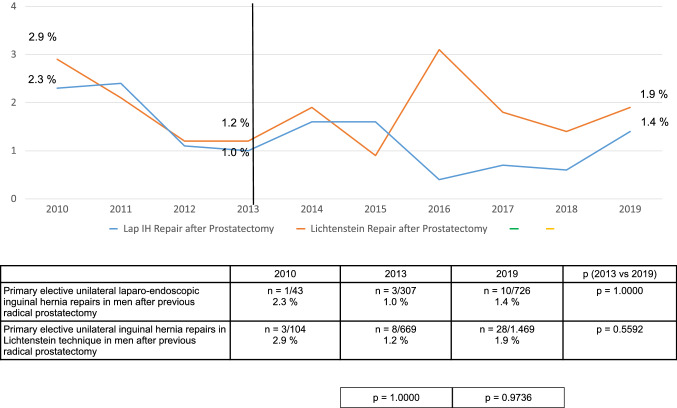
Complication-related reoperation rates of primary elective unilateral inguinal hernia repairs in men in laparo-endoscopic or Lichtenstein technique following radical prostatectomy (2010–2019) (*n* = 12,465)

This trend in the perioperative findings was observed relatively consistently for the years 2013–2019 (Figs. 3, 4, 5). Details of 1-year follow-up were available for 80.1% (*n* = 214,341/267,687) of patients.

Comparison of primary elective unilateral inguinal hernia repair in men with and without previous radical prostatectomy did not find any significant differences in the recurrence rates for either the laparo-endoscopic techniques or the open Lichtenstein technique.

Matters were different for the pain rates. Following primary elective unilateral inguinal hernia repair in men after previous radical prostatectomy in laparo-endoscopic technique, a significantly lower rate of pain at rest (3.2% vs 4.2%; *p* = 0.010) and pain on exertion (5.8% vs 8.3%; *p* = 0.010) was identified compared with the patients without previous prostatectomy (Table 5). Likewise, for the open Lichtenstein technique after previous radical prostatectomy a significantly lower rate of pain on exertion (7.2% vs 8.3%; *p* = 0.003) was found compared with the patients without previous radical prostatectomy.

**Table 5:** Pain at rest, pain on exertion, pain requiring treatment and recurrence rates of primary elective unilateral inguinal hernia repair in men in TEP, TAPP and Lichtenstein technique with and without previous radical prostatectomy at 1-year follow-up (2010–2019) (*n* = 214,341)

	Pain at rest	Pain on exertion	Pain requiring treatment	Recurrence rate
Primary elective unilateral laparo-endoscopic inguinal hernia repairs in men without previous radical prostatectomy	*n* = 5.769/136.731 4.2%	*n* = 11.352/136.731 8.3%	*n* = 3.271/136.731 2.4%	*n* = 1.292/136.731 0.9%
Primary elective unilateral laparo-endoscopic inguinal hernia repairs in men after previous radical prostatectomy	*n* = 107/3.299 3.2%	*n* = 191/3.299 5.8%	*n* = 69/3.299 2.1%	*n* = 34/3.299 1.0%
	*p* = 0.0098	*p* < 0.001	*p* = 0.5964	*p* = 1.000
Primary elective unilateral inguinal hernia repairs in Lichtenstein technique in men without previous radical prostatectomy	*n* = 2.886/67.127 4.3%	*n* = 5.581/67.127 8.3%	*n* = 1.571/67.127 2.3%	*n* = 505/67.127 *n* = 0.8%
Primary elective unilateral inguinal hernia repairs in Lichtenstein technique in men after previous radical prostatectomy	*n* = 285/7.184 4.0%	*n* = 520/7.184 7.2%	*n* = 142/7.184 2.0%	*n* = 54/7.184 0.8%
	*p* = 0.3940	*p* = 0.0028	*p* = 0.1036	*p* = 1.000

Direct comparison of primary elective unilateral inguinal hernia repair in men after previous radical prostatectomy identified for patients operated on with the laparo-endoscopic technique a significantly lower rate of pain on exertion (5.8% vs 7.2%; *p* = 0.013) than those operated on with the open Lichtenstein technique (Table 6). Comparison of the operation times show a mean value of 59 min for the laparo-endoscopic repair and 58 min for the open Lichtenstein technique after previous radical prostatectomy.

**Table 6:** Pain at rest, pain on exertion, pain requiring treatment and recurrence rates of primary elective unilateral inguinal hernia repair in men in TEP, TAPP and Lichtenstein technique after previous radical prostatectomy at 1-year follow-up (2010–2019) (*n* = 10,483)

	Pain at rest	Pain on exertion	Pain requiring treatment	Recurrence rate
Primary elective unilateral laparo-endoscopic inguinal hernia repairs in men after previous radical prostatectomy	*n* = 107/3.299 3.2%	*n* = 191/3.299 5.8%	*n* = 69/3.299 2.1%	*n* = 34/3.299 1.0%
Primary elective unilateral inguinal hernia repairs in Lichtenstein technique in men after previous radical prostatectomy	*n* = 285/7.184 4.0%	*n* = 520/7.184 7.2%	*n* = 142/7.184 2.0%	*n* = 54/7.184 0.8%
	*p* = 0.1520	*p* = 0.0130	*p* = 1.000	*p* = 0.3322

## Discussion

This present analysis of data from the Herniamed Registry demonstrates that a proportion of 4.7% of all patients with primary elective unilateral inguinal hernia repair in men had undergone previous radical prostatectomy.

Two-thirds of hernias occurred after open and one-third after minimally invasive radical prostatectomy. Throughout the study period from 2010 to 2019 two-thirds of all primary elective unilateral inguinal hernia repairs in men after previous radical prostatectomy were carried out relatively consistently in open Lichtenstein technique and one-third in laparo-endoscopic technique.

For primary elective unilateral inguinal hernia repair in men with and without previous radical prostatectomy, the laparo-endoscopic techniques after previous radical prostatectomy were found to have significantly higher rates of intra- and postoperative complications as well as complication-related reoperations.

Further analysis revealed for the intraoperative complications after previous radical prostatectomy significantly more bladder and vascular injuries for TEP and TAPP. There were more cases of postoperative secondary bleeding and seroma.

On comparing primary elective unilateral inguinal hernia repair in men with and without previous radical prostatectomy for the Lichtenstein technique only a significantly higher rate of secondary bleeding after previous radical prostatectomy was identified.

Direct comparison of the TEP and TAPP surgical techniques versus the Lichtenstein technique for primary elective unilateral inguinal hernia repair in men with previous radical prostatectomy demonstrated for the laparo-endoscopic techniques a significantly higher intraoperative complication rate, and for the open Lichtenstein technique a significantly higher rate of postoperative complications and complication-elated reoperations. This trend was consistently identified over the years 2013–2019. Hence, there are no clear advantages for one or the other technique. The surgeon can opt for a particular surgical technique based on their own personal experience.

Neither for the laparo-endoscopic techniques nor the open Lichtenstein technique for primary elective unilateral inguinal hernia repair in men with previous radical prostatectomy found to have an unfavorable influence on the pain or recurrence rates at 1-year follow-up. On the contrary, the pain rates following primary elective unilateral inguinal hernia repair in men after previous radical prostatectomy were found to be even significantly lower. This may be due to the fact that nerves to the inguinal region had already been transected during the previous radical prostatectomy.

Registry studies have special limitations. Participation in the Herniamed Registry is voluntary. Hence, not all hospitals and surgeons from the participating countries take part. This could represent a certain bias. The completeness and correctness of data can be checked by the auditors only at the time of certification of hernia centers. No follow-up is available for a relevant proportion of all patients. Follow-up is performed by sending a questionnaire to the patient and his general practitioner and selective physical examination by the treating hospital/surgeon or the general practitioner. Therefore, the follow-up findings must be interpreted with caution.

In summary, it can be stated that around 30% of inguinal hernias after previous radical prostatectomy are repaired using a laparo-endoscopic technique. When comparing the perioperative outcome of primary elective unilateral inguinal hernia repair in men with and without previous radical prostatectomy, significantly higher rates of perioperative complications were identified for the laparo-endoscopic techniques and for the Lichtenstein technique no significant differences were found because of the previous radical prostatectomy. Direct comparison of the outcome of primary elective unilateral inguinal hernia repair in men after previous radical prostatectomy identified for the laparo-endoscopic techniques significantly more intraoperative complications and for the open Lichtenstein technique significantly more postoperative complications and complication-related reoperations. Hence, since there are no clear advantages for one or the other surgical procedure, which surgical technique is chosen should depend on the surgeon’s experience.
